# A p53-independent role of Mdm2 in estrogen-mediated activation of breast cancer cell proliferation

**DOI:** 10.1186/bcr2804

**Published:** 2011-01-11

**Authors:** Angelika Brekman, Kathryn E Singh, Alla Polotskaia, Nandini Kundu, Jill Bargonetti

**Affiliations:** 1 Department of Biological Sciences, Hunter College and The Graduate Center Biochemistry and Biology Programs, CUNY, 695 Park Ave, New York, NY 10065, USA

## Abstract

**Introduction:**

Estrogen receptor positive breast cancers often have high levels of Mdm2. We investigated if estrogen signaling in such breast cancers occurred through an Mdm2 mediated pathway with subsequent inactivation of p53.

**Methods:**

We examined the effect of long-term 17β-estradiol (E2) treatment (five days) on the p53-Mdm2 pathway in estrogen receptor alpha (ERα) positive breast cancer cell lines that contain wild-type *p53 *(MCF-7 and ZR75-1). We assessed the influence of estrogen by examining cell proliferation changes, activation of transcription of p53 target genes, p53-chromatin interactions and cell cycle profile changes. To determine the effects of Mdm2 and p53 knockdown on the estrogen-mediated proliferation signals we generated MCF-7 cell lines with inducible shRNA for *mdm2 *or *p53 *and monitored their influence on estrogen-mediated outcomes. To further address the p53-independent effect of Mdm2 in ERα positive breast cancer we generated cell lines with inducible shRNA to *mdm2 *using the mutant p53 expressing cell line T-47D.

**Results:**

Estrogen increased the Mdm2 protein level in MCF-7 cells without decreasing the p53 protein level. After estrogen treatment of MCF-7 cells, down-regulation of basal transcription of p53 target genes *puma *and *p21 *was observed. Estrogen treatment also down-regulated etoposide activated transcription of *puma*, but not *p21*. Mdm2 knockdown in MCF-7 cells increased p21 mRNA and protein, decreased cell growth in 3D matrigel and also decreased estrogen-induced cell proliferation in 2D culture. In contrast, knockdown of p53 had no effect on estrogen-induced cell proliferation. In T-47D cells with mutant p53, the knockdown of Mdm2 decreased estrogen-mediated cell proliferation but did not increase p21 protein.

**Conclusions:**

Estrogen-induced breast cancer cell proliferation required a p53-independent role of Mdm2. The combined influence of genetic and environmental factors on the tumor promoting effects of estrogen implicated Mdm2 as a strong contributor to the bypass of cell cycle checkpoints. The novel finding that p53 was not the key target of Mdm2 in the estrogen activation of cell proliferation could have great benefit for future Mdm2-targeted breast cancer therapies.

## Introduction

While the *p53 *gene is the most commonly mutated gene in human cancers [1], *p53 *mutations in breast cancers occur in only 20% of cases [2-4]. Breast cancer cells with wild-type *p53 *often have high levels of the oncogenic protein Mdm2 suggesting that Mdm2 might block the function of p53 [5-7]. In addition, elevated expression of Mdm2 occurs in estrogen receptor α positive (ERα +) breast cancer cells independently of p53 using evolutionarily conserved AP1 and ETS family transcription factors [8]. Two-thirds of breast cancers demonstrate estrogen-dependent growth [9]. In response to estrogen, ERα induces transcription of target genes to activate cell proliferation and survival [10,11]. This could be, in part, through coordinated activation of cell proliferation and inhibition of cell death. Estrogen induces expression of the anti-apoptotic gene *bcl-2 *thus inhibiting apoptosis [12] and also stimulates Myc expression to aid in cell survival [11]. In addition, estrogen can influence the p53-pathway because ERα can inhibit p53 transcriptional activity by interacting with p53 on the chromatin [13,14]. While Mdm2 has been implicated in estrogen's mechanism of action, the role that Mdm2 plays in this process has not been clearly defined [6,15-17]. Mdm2 expression is increased in the presence of estrogen [8,18] and Mdm2 enhances the function of ERα [19]. A cancer predisposition single nucleotide polymorphism at position 309 in the *mdm2 *gene P2 promoter (T→G) increases binding affinity for the SP1 transcription factor, leading to Mdm2 over-expression [20]. The Mdm2 SNP309 G allele has also been associated with inhibition of the p53-Mdm2 pathway [20,21]. In cancer cells that are homozygous G/G for the Mdm2 SNP309, the Mdm2 protein remains associated with the chromatin as a p53-Mdm2 complex resulting in compromised p53 trans-activation [21]. Moreover, the *mdm2 *SNP309 G allele associates with accelerated tumor formation in a gender-specific and hormone-dependent manner [6]. Therefore, the connection between estrogen and Mdm2 implicates inhibition of p53 as a possible mechanism of action.

In normal cells *mdm2 *transcription is activated by p53 [22] and Mdm2 protein functions to target p53 for proteolysis [23,24]. Mdm2 also inhibits p53 transactivation by blocking p53 association with the transcription machinery [25-28]. Additionally, Mdm2 mediates histone ubiquitination leading to repression of p53 targets [29]. Mdm2 has also been shown to target p21 for proteasomal turnover independently of ubiquitination [30]. Mdm2 may impart some of its tumorigenic properties by increasing the degradation of multiple cellular proteins.

If Mdm2 is blocking the p53-pathway in estrogen receptor positive breast cancer cells then the conventional chemotherapeutics that rely on the p53 tumor suppressor, a major cell death regulator [31], may not activate cell death effectively. p53 acts by promoting expression of numerous genes which control cell cycle arrest, senescence, apoptosis, DNA repair, genomic stability and survival [32]. p53 also plays a pro-apoptotic role by activating target genes that produce products that dimerize with Bcl-2, one critical target of this type is *puma *[33]. Endocrine therapy is used in ERα+ breast cancers and this reduces Bcl-2 levels, however, due to acquired resistance other treatment options need to be identified [34]. The involvement of the p53-Mdm2 pathway in estrogen's influences places this pathway at the forefront of our investigation.

Using inducible gene silencing of *mdm2 *and *p53 *we examined if the p53-Mdm2 pathway was required for estrogen-mediated cell proliferation. We found that a p53-independent role for Mdm2 participated in estrogen-induced proliferation of MCF-7 and T-47D breast cancer cells. Inducible knockdown of Mdm2 in MCF-7 cells with wild-type p53 decreased cell proliferation and increased p21. Moreover, inducible gene silencing of *mdm2 *caused a reduction in the estrogen-induced target Bcl-2. Inducible knockdown of Mdm2 in estrogen treated T-47D cells with oncogenic mutant p53 decreased cell proliferation without increasing p21. Our data suggest that estrogen activates cell proliferation using Mdm2 to repress multiple cell cycle checkpoints as evidenced by comparison of MCF-7 and T-47D outcomes following inducible shRNA mediated knockdown of Mdm2.

## Materials and methods

### Cell culture

MCF-7 (*p53 *wild-type, *mdm2 *SNP309 T/G), T-47D (oncogenic mutant p53 L194F, *mdm2 *SNP309 G/G) and ZR75-1 (*p53 *wild-type, *mdm2 *SNP309 T/T) from American Type Culture Collection (ATCC). MCF-7 and ZR75-1 cells were grown in RPMI 1640 medium (Mediatech) and T-47D cells were grown in DMEM medium (Invitrogen, Carlsbad, CA, USA). Both media were supplemented with 10% FBS (Gemini, West Sacramento, CA, USA) and 2,500 units of penicillin-streptomycin (Mediatech, Herndon, VA, USA) at 5% CO_2 _37°C humidified incubator. We generated constructs with inducible (TET-ON) shRNA for *mdm2 *and *p53 *or without the shRNA oligonucleotide (a generous gift from Scott Lowe). Constructs were introduced into the MCF-7 cells (*mdm2 *or *p53 *shRNA) and T-47D cells (*mdm2 *shRNA) by retrovirus mediated gene transfer method. Briefly, Phoenix packaging cells were transfected by calcium phosphate method with either an rtTA plasmid or with a vector containing *mdm2*, *p53 *or no shRNA oligo. The generated viruses were harvested and MCF-7 cells or T-47D cells were co-infected with the rtTA plasmid and one of the vectors. After selection with puromycin (vector with shRNA) and hygromycin (rtTA), clonal cell lines were generated by limited dilution method. Clonal cell lines were selected based on the level of Mdm2 or p53 knockdown. Experiments shown were carried out on clonal cell lines. MCF-7 *mdm2 *shRNA 151656 clone C4; T-47D *mdm2 *shRNA 151657 clone 3B6; MCF-7 *p53 *shRNA 2120 clone D11. To induce shRNA expression, cells were treated with 2 μg/ml doxycycline (DOX) for time periods indicated in the figures.

### Treatments

Estrogen (17β-estradiol, E2), Etoposide and DMSO were purchased from Sigma, Saint Louis, MO, USA. 24 hours prior to treatments, growth medium was changed to phenol-red-free RPMI 1640 (for MCF-7 cells) or DMEM (for T-47D cells) containing 10% charcoal-stripped FBS (Gemini) and antibiotics. Fresh medium was supplemented every 72 hours.

### Quantitative reverse transcription-PCR (qRT-PCR)

RNA was isolated using QIAshredder columns and RNeasy Mini Kit (Qiagen, Valencia, CA, USA). A total of 5 μg of RNA was used for cDNA synthesis using High Capacity cDNA Archive Kit reagents (Applied Biosystems, Foster City, CA, USA). 150 ng of cDNA was combined with Taqman Universal Master Mix (Applied Biosystems, Foster City, CA, USA) and Applied Biosystems Assays on Demand primers/probes for *puma *(Hs00248075_m1)*, mdm2 *(Hs00242813_m1)*, p21 *(Hs00355782_m1) or *ACTIN *(4352935E). PCR reaction was carried out in 7500 Sequence Detection System (Applied Biosystems). *P*-values were calculated by student *t*-test.

### Whole cell protein extract

Cells were lysed in RIPA buffer (0.1% SDS, 1% NP-40, 0.5% Deoxycholate, 150 mM NaCl, 1 mM EDTA, 0.5 mM EGTA, 50 mM Tris-Cl pH8) with 1 mM PMSF, 8.5 μg/ml Aprotinin and 2 μg/ml Leupeptin following standard protocol.

### Western blot

A total of 50 μg of protein extract were separated by 10% SDS-PAGE and electro-transferred to nitrocellulose membrane. Immunoblotting was done with p53 antibodies (a 1:1:1 mix of hybridoma supernatants, pAb421, pAb240 and pAb1801); Mdm2 in MCF-7 and ZR75-1 cells (SMP-14 Santa Cruz sc-965, Santa Cruz Biotechnology, Santa Cruz, CA, USA); Mdm2 in T-47D cells (a 1:1:1 mix of hybridoma supernatants, 4B2, 2A9 and 4B11); Bcl-2 (100 Santa Cruz sc-509); PUMA (Cell Signaling 4976, Danvers, MA, USA); p21 (Ab-1 Oncogene Research Science OP64, Gibbstown, NJ, USA); Actin (Sigma A2066). To detect p21 protein in T-47D cells, 100 μg of protein extract was transferred to a PVDF membrane.

### Immunofluorescence

Cells, grown and treated on coverslips, were fixed with 4% Formaldehyde and permeabilized with 0.5% Triton-X-100. Immunohistochemistry was done with p53 (FL-393 Santa Cruz sc-6243) and Mdm2 (SMP-14 Santa Cruz sc-965) antibodies followed by incubation with FITC-conjugated anti-mouse (Jackson ImmunoResearch 715-095-150, West Grove, PA, USA) and Alexa-conjugated anti-rabbit (Invitrogen A11037). Coverslips were mounted onto slides using Vectashield mounting medium with DAPI (Fisher Scientific NC9524612, Pittsburgh, PA, USA). Images were collected by PerkinElmer UltraVIEW ERS Spinning Disc Microscope, Waltham, MA, USA.

### Chromatin immunoprecipitation (ChIP)

Cells were incubated with 1% Formaldehyde for 30 minutes at 5% CO_2 _37°C humidified incubator, followed by 0.125 M Glycine treatment for 5 minutes. Cells were lysed in RIPA buffer with 1 mM PMSF, 8.5 μg/ml Aprotinin, 2 μg/ml Leupeptin and Phosphatase Inhibitor Cocktail 1 (Sigma)). Lysates were sonicated 10 times (one minute pulse and one minute rest) in a Branson Digital Sonifier, Danbury, CT, USA and spun down for 30 minutes 13,000 rpm at 4°C. 400 μg of protein from cell lysates were subjected to overnight incubation at 4°C with 2 μg of p53 (Ab-6 Calbiochem OP43, Gibbstown, NJ, USA); Mdm2 (N-20 Santa Cruz sc-813); or non-specific IgG (Santa Cruz, IgG mouse sc-2025, IgG rabbit sc-2027). 50 μl of 25% beads slurry of protein A/G Plus Agarose beads (Santa Cruz sc-2003), pre-blocked with 0.3 mg/ml sheared herring sperm DNA (Invitrogen, 15634-017), were added to immunoprecipitation samples for two hours at 4°C, followed by washes: *(1) *0.1% SDS, 1% Triton-X-100, 20 mM Tris pH8.1, 150 mM NaCl; *(2) *0.1% SDS, 1% Triton-X-100, 20 mM Tris pH8.1, 500 mM NaCl; *(3) *0.25 M LiCl, 1% NP-40, 1% Deoxycholate, 1 mM EDTA, 10 mM Tris pH8; and *(4) *twice with TE pH8. Immunoprecipitated chromatin was de-crosslinked overnight at 65°C with 1 mg/ml ProteinaseK, 1% SDS and 0.1M NaHCO_3_. For total DNA input, 40 μg were similarly de-crosslinked. DNA fragments were purified using Qiagen QiaQuick kit (Qiagen) and amplified by real-time quantitative PCR in 7500 Sequence Detection System (Applied Biosystems). Primers and probes sequences are based on [35], and are provided below:

puma:

forward primer: GCGAGACTGTGGCCTTGTGT;

reverse primer: CGTTCCAGGGTCCACAAAGT;

probe: TGTGAGTACATCCTCTGGGCTCTGCCTG.

mdm2:

forward primer: GGTTGACTCAGCTTTTCCTCTTG;

reverse primer: GGAAAATGCATGGTTTAAATAGCC;

probe: GCTGGTCAAGTTCAGACACGTTCCGAA.

p21:

forward primer: GTGGCTCTGATTGGCTTTCTG;

reverse primer: CTGAAAACAGGCAGCCCAA;

probe: TGGCATAGAAGAGGCTGGTGGCTATTTTG.

### *mdm2 *siRNA transfection

Cells were seeded in media with no antibiotics. After 24 hours, 10 μl Lipofectamine2000 (Invitrogen) was incubated for 5 minutes with 240 μl Optimem (Invitrogen). 0.2 nmol (100 nM) of non-specific or *mdm2 *siRNA (Dharmacon) were resuspended in 250 μl Optimem and combined with Lipofectamine2000. After 20 minutes, 500 μl siRNA-Lipofectamine2000 mix was added to cells with 1.5 ml Optimem. Six hours later, complete growth media was supplemented.

### Cell proliferation

Number of cells was determined by the Guava Viacount assay according to manufacturer's protocol (Millipore, Lincoln Park, NJ, USA). Graphs show means and standard errors of three independent experiments. P-values were calculated by student t-test.

### Fluorescence activated cell sorting (FACS)

FACS was performed on a FACScan (BD Biosciences, San Jose, CA, USA). After treatments, cells were harvested, washed, resuspended in PBS containing 2% bovine serum albumin, 0.1% sodium azide, fixed in 30% ethanol, and stored overnight at 4°C. Before sorting, propidium iodide staining and RNase treatment were performed for 30 minutes at 37°C.

### Cell culture in matrigel

MCF-7 cells were seeded at a density of 5 × 10^3 ^cells per chamber in an eight chamber slide on top of 50 μl solidified matrigel (BD Biosciences) in MEBM basal medium without phenol red (Lonza CC-3153, Walkersville, MD, USA) supplemented with bullet kit components except for BPE (Lonza CC-4156), 10% charcoal FBS and 2% matrigel, in the presence of 10 nM estrogen and in the absence or presence of 2 μg/ml doxycycline. Medium was changed every three days. Brightfield pictures show mass structures that MCF-7 cells form in matrigel after three weeks. MCF-7 cells were also fixed directly in culture with 4% Formaldehyde and stained with propidium iodide. Confocal analysis was performed using Laser scanning spectral confocal microscope TCS SP2. Large, intermediate and small mass structures were counted and presented as percent of the total population.

## Results

### Estrogen perturbs the p53-Mdm2 pathway in breast cancer cells

**Figure 1:**
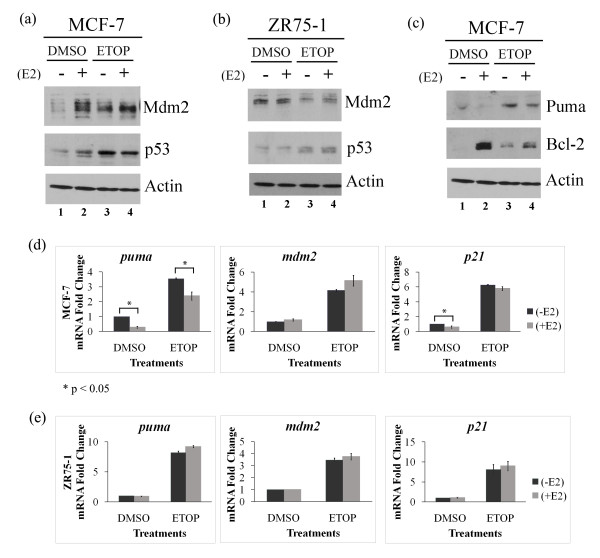
**Estrogen provokes increased Mdm2 and compromised transactivation of the p53 apoptotic target *puma***. MCF-7 and ZR75-1 cells were treated with 10 nM estrogen (E2) for five days and 50 μM etoposide (ETOP) for 48 hours. Western blot analysis of p53, Mdm2, and Actin protein levels from whole cell lysates of MCF-7 **(a) **and ZR75-1 **(b) **cells respectively. **(c) **Protein levels of Puma, Bcl-2 and Actin from whole cell lysates analyzed by Western blot. Quantitative real-time PCR (RT-PCR) was carried out for mRNA levels of *puma, mdm2 *and *p21 *genes in MCF-7 **(d) **and ZR75-1 **(e) **cells respectively. Results were normalized to control samples and actin values. Graphs show means and standard errors of two independent experiments. * *P *< 0.05 (Student *t*-test with * to indicate estrogen significantly influenced the transactivation of a p53 target gene).

Estrogen receptor α positive (ERα+) breast cancer cells often have wild-type *p53 *and estrogen stimulates proliferation of these cells [2]. Protein levels of p53 and its negative regulator Mdm2 normally oscillate [36]. This oscillation is perturbed in MCF-7 breast cancer cells that carry the *mdm2 *SNP309 T to a G change (T→G) but is not perturbed in the breast cancer cell line ZR75-1 that does not have this change [15]. Moreover, a major survival advantage is seen in MCF-7 cells treated with estrogen for five days as compared to 24 hours [11,37,38]. Therefore, we examined the effect of five days of estrogen treatment on the p53-dependent signal transduction in MCF-7 and ZR75-1 cells. In MCF-7 cells, Mdm2 protein increased after five days of estrogen treatment while surprisingly, p53 protein was not decreased and showed a slight increase (Figure 1a, lanes 1 and 2). However, estrogen decreased basal levels of *puma *and *p21 *transcripts (Figure 1d). To determine if estrogen influenced p53-dependent signal transduction after DNA damage, we treated the cells with the chemotherapeutic drug etoposide. DNA damage by etoposide increased the p53 protein level in the MCF-7 cells while estrogen treatment caused no further increase (Figure 1a, compare lanes 1 and 3 and lanes 3 and 4, for p53). Etoposide treatment also increased the Mdm2 protein level in MCF-7 cells and estrogen treatment for five days caused little further increase (Figure 1a, compare lanes 1 and 3 and lanes 3 and 4, for Mdm2). When we examined the p53 transcriptional activity after drug treatment we saw a statistically significant reduction in the etoposide-mediated activation of the *puma *gene after estrogen treatment however transcription of the target genes *mdm2 *and *p21 *was not changed (Figure 1d).

We also examined the influence of estrogen on the p53-dependent signal transduction in ZR75-1 cells that have been shown to have intact oscillations for p53 and Mdm2 [15]. The addition of estrogen did not influence the Mdm2 or p53 levels in these cells (Figure 1b, lanes 1-4, p53 and Mdm2). We saw that p53 levels increased following DNA damage by the drug etoposide in ZR75-1 cells (Figure 1b, lanes 3 and 4) and that estrogen did not influence the etoposide-mediated activation of p53 target genes (Figure 1e).

In order to study the influence of estrogen-mediated outcomes driven through upregulation of Mdm2 we focused our attention on the MCF-7 cell line. Since estrogen decreased transcription of the p53 pathway target *puma *following chemotherapeutic treatment with etoposide, we examined if PUMA protein levels were also reduced. In direct correspondence to the transcription data (Figure 1d) PUMA protein was reduced following estrogen treatment and this was also seen when p53 was activated by etoposide treatment of MCF-7 cells (Figure 1c, compare lanes 1 and 2 and lanes 3 and 4, for PUMA). In addition, a coordinate increase in the anti-apoptotic protein Bcl-2 was seen following estrogen treatment (Figure 1c, compare lanes 1 and 2 and lane 3 and 4, for Bcl-2).

### p53 and Mdm2 nuclear localization and chromatin association are not influenced by estrogen treatment

**Figure 2:**
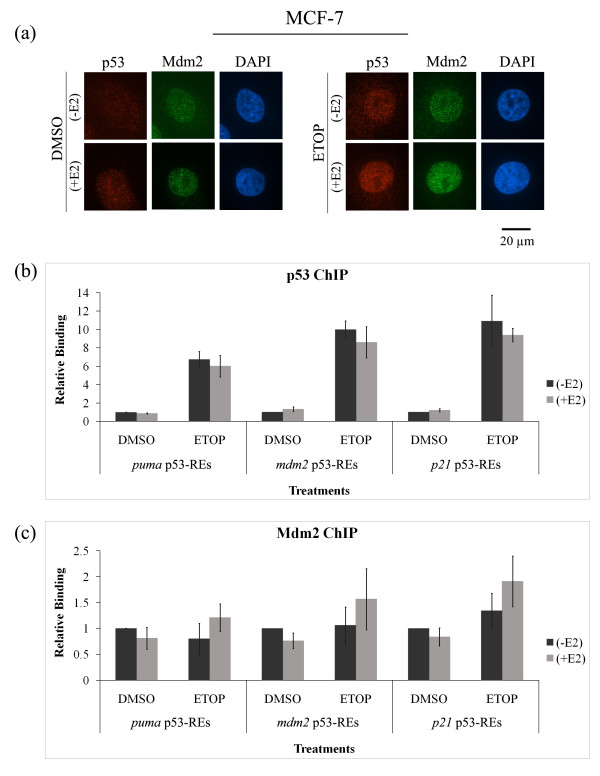
**p53 and Mdm2 nuclear localization and chromatin association are not influenced by estrogen treatment**. MCF-7 cells were treated with 10 nM estrogen (E2) for five days and 50 μM etoposide (ETOP) for 48 hours. **(a) **Immunofluorescence was carried out for p53 and Mdm2 proteins. Nuclear DNA was stained with DAPI. 400 μg of cross-linked and sonicated whole cell lysates were subjected to chromatin immunoprecipitation (ChIP), using antibodies against p53 **(b) **or Mdm2 **(c)**. ChIP with non-specific IgG was done to subtract the background. Immunoprecipitated DNA was amplified by real-time quantitative PCR with primers and FAM-labeled probes for p53-REs in *puma*, *mdm2 *and *p21 *genes. Values were normalized to IgG and inputs, followed by normalization to the DSMO samples. Graphs show means and standard errors of three independent experiments. The changes in relative binding of p53 and Mdm2 to the three different p53-REs were not significant (*P *> 0.05). The *P*-values calculated by student T-test for the difference between with or without E2 were all between 0.1 and 0.25.

Nuclear exclusion of p53 has been documented to occur following estrogen treatment of breast cancer cells [39,40]. We therefore evaluated if nuclear exclusion of p53 could explain any of the estrogen-mediated changes we saw in MCF-7 cells. We examined the effect of estrogen treatment on the cellular localization of p53 and Mdm2 (Figure 2a). Localization of p53 was unchanged by estrogen treatment of MCF-7 cells and the Mdm2 in the same cells was predominantly localized to the nuclei (Figure 2a, DMSO representative nuclei shown). When the cells were treated with etoposide to damage the DNA and activate the p53 pathway the p53 level increased in the nuclei and estrogen did not block p53 protein localization (Figure 2a, ETOP representative nuclei shown). Both Mdm2 and p53 were evident in punctate nuclear foci before and after estrogen treatment.

To determine if estrogen decreased the ability of nuclear p53 protein to interact with p53-responsive elements (p53-REs) of target genes we carried out quantitative chromatin immunoprecipitation (ChIP) experiments and examined p53 recruitment to the p53-REs of *puma*, *mdm2 *and *p21 *genes in MCF-7 cells. Following etoposide treatment, p53 was recruited to the p53-REs of *puma*, *mdm2 *and *p21 *genes (Figure 2b). Estrogen treatment did not significantly decrease the p53-chromatin interaction at any of the p53-REs. A trend towards increased Mdm2 protein recruitment on the chromatin was observed following etoposide plus estrogen treatment, however this change was not statistically significant (Figure 2c).

### Transactivation of p53 target genes following *mdm2 *gene silencing

**Figure 3:**
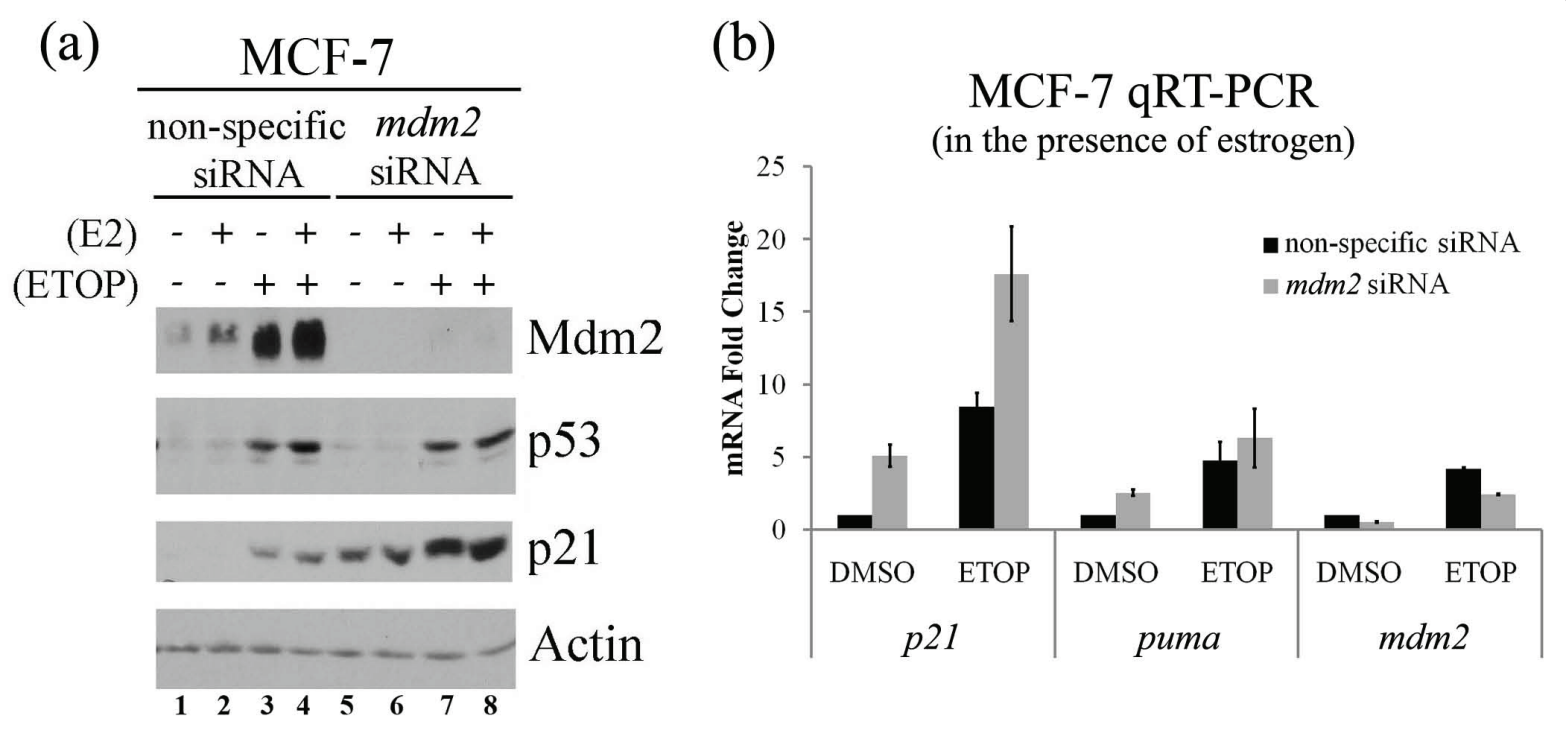
**Gene silencing of *MDM*2 by siRNA potentiates p53 transactivation in the presence of estrogen**. MCF-7 cells were transfected with 100 nM non-specific or *mdm2 *siRNA. 24 hours following transfection, cells were treated with 10 nM estrogen (E2) for 24 hours and 50 μM etoposide (ETOP) for 3 hours. **(a) **Western blot analysis of Mdm2, p53, p21 and Actin protein levels from whole cell lysates. **(b) **Quantitative real-time RT-PCR for mRNA levels of *puma, p21 *and *mdm2 *genes in MCF-7 cells. Results were normalized to control samples and *actin *values. Graphs show means and standard errors of two independent experiments.

We predicted that the estrogen elicited growth promoting effects resulted in part through the increase in Mdm2 and therefore reasoned that decreased *mdm2 *expression would translate into reduced estrogen associated molecular changes in the cells. Because of the documented relationship between Mdm2 and p53 we continued to examine this pathway. Mdm2 was knocked down by siRNA to determine if any of the molecular outcomes resulting from estrogen treatment required Mdm2. The addition of siRNA to *mdm2 *was very effective at reducing Mdm2 protein levels in estrogen and etoposide treated cells (Figure 3a, compare lanes 1-4 to lanes 5-8, for Mdm2). Knockdown of *mdm2 *by siRNA did not increase the p53 protein level in the absence or presence of etoposide (Figure 3a, compare lanes 1-4 to lanes 5-8, for p53). However, knockdown of *mdm2 *by siRNA caused a dramatic increase in the level of p21 protein in the presence or absence of estrogen (Figure 3a, compare lanes 1-4 to lanes 5-8, for p21). Knockdown of *mdm2 *also resulted in increased expression of *p21 *and *puma *mRNA as determined by quantitative RT-PCR (Figure 3b). This suggests that while p53 protein did not increase following Mdm2 knockdown, the p53-dependent signal transduction pathway was activated. The increased p21 protein observed could therefore result from both p53-dependent regulation of transcription and p53-independent regulation by loss of Mdm2-mediated p21 degradation.

### Inducible Mdm2 knockdown inhibits estrogen-mediated MCF-7 and T-47D cell proliferation

**Figure 4:**
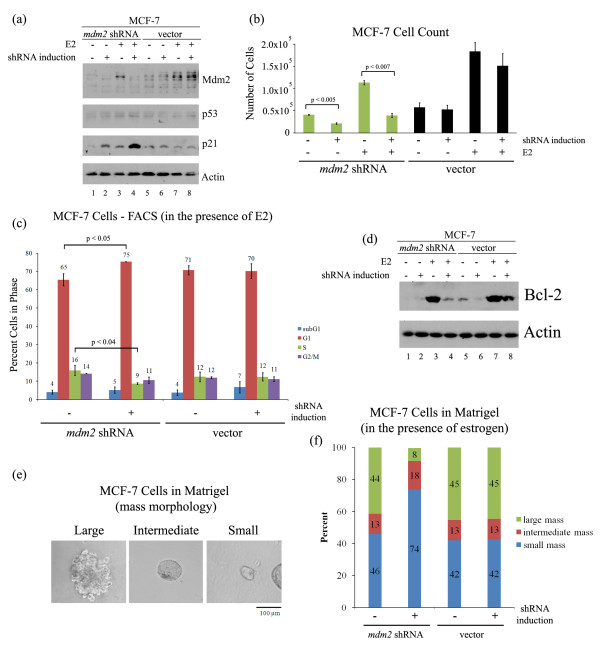
**Mdm2 knockdown in estrogen-treated MCF-7 cells inhibits cell proliferation**. Clonal MCF-7 cell lines with *mdm2 *shRNA or control vector were treated with 2 μg/ml doxycycline (DOX) for three days to induce shRNA expression, followed by 10 nM estrogen (E2) for five days in the presence of DOX. **(a) **Western blot analysis of Mdm2, p53, p21 and Actin protein levels from whole cell lysates. **(b) **Number of cells was determined by Guava Viacount assay. 10,000 cells were seeded at beginning of treatments. **(c) **Fluorescence activated cell sorting (FACS). Cells were harvested, fixed in 30% ethanol, and cellular DNA was stained with propidium iodide. **(d) **Western blot analysis of Bcl-2 and Actin protein levels from whole cell lysates. **(e) **MCF-7 cells, grown in matrigel for three weeks, formed mass structures of three sizes: large, intermediate and small. **(f) **MCF-7 cells, grown in matrigel for three weeks in the presence of 10 nM estrogen and in the absence or presence of 2 μg/ml doxycycline, were fixed and stained with propidium iodide. Masses of different sizes (large, intermediate and small) were counted and presented as percent of the total population. On average, a total of 300 masses were counted. Averages of three independent experiments are shown. The significance in the percent change of large and small masses was determined by the Student *t*-test (*P *< 0.05).

To determine the contribution of Mdm2 or p53 to the estrogen-induced cell proliferation, we generated inducible short-hairpin RNA constructs (shRNAs) targeting *mdm2 *or *p53 *using the mir-30 design (a generous gift from Agustin Chicas and Scott Lowe). These shRNA constructs and control constructs were transduced into the MCF-7 cells and stable clones were selected. As shown in Figure 4a induction of *mdm*2 shRNA reduced Mdm2 protein while similar induction of the control vector construct did not (Figure 4a, compare lanes 2 and 4 to lanes 6 and 8, for Mdm2). The induction of *mdm2 *shRNA also resulted in a dramatic increase in p21 protein (Figure 4a, compare lanes 2 and 4 to lanes 6 and 8, for p21). This was observed for multiple clones as well as a selected pool (data not shown).

Induced knockdown of *mdm2 *repressed estrogen-mediated cell proliferation, while the control vector showed no change (Figure 4b). When the cell cycle profile was examined we saw a reduction in S phase cells and an increase in G1 phase populations following Mdm2 knockdown (Figure 4c). No increase in cell death was detected following Mdm2 knockdown (data not shown), but we did see a decrease in the estrogen-induced anti-apoptotic protein Bcl-2 (Figure 4d, compare lanes 4 and 8).

In addition to examining the role of Mdm2 in cell proliferation in 2D culture, we also examined the effect of Mdm2 knockdown on estrogen treated MCF-7 cell proliferation in matrigel (3D culture). Normal mammary epithelial cells cultured in matrigel organize into polar, acini-like structures, while mammary tumor cells continue to proliferate into disorganized masses [41] that correlate with their gene expression profiles [42]. We observed that MCF-7 cells grown in matrigel for three weeks in the presence of estrogen formed masses of three different sizes: large, intermediate and small (Figure 4e). The intermediate mass structure resembled the acinus in shape and size, but had a filled lumen, as reported by Bissell laboratory [42] and data not shown. Only a small percent of the structures (about 13%) had an intermediate mass size, while about 42-45% of the structures were either large or small (Figure 4f). Interestingly, Mdm2 knockdown led to a substantial decrease in the number of large structures and an increased number of small structures (Figure 4f).

**Figure 5:**
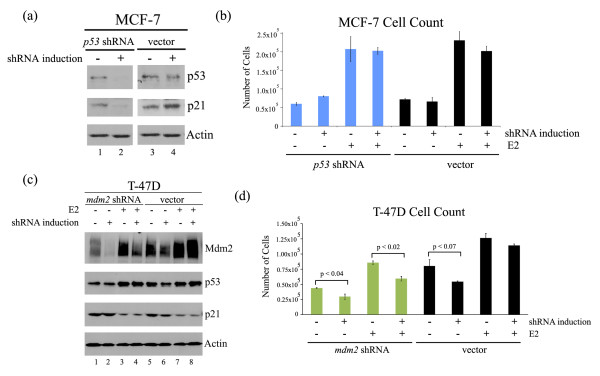
**p53 knockdown in estrogen-treated MCF-7 cells does not promote cell proliferation and Mdm2 knockdown in estrogen-treated T-47D cells decreases cell proliferation**. Clonal MCF-7 cell lines with *p53 *shRNA or control vector were treated with 2 μg/ml doxycycline (DOX) for three days to induce shRNA expression, followed by 10 nM estrogen (E2) for five days in the presence of DOX. **(a) **Western blot analysis of p53, p21 and Actin protein levels from whole cell lysates. **(b) **Number of cells was determined by Guava Viacount assay. 10,000 cells were seeded at beginning of treatments. **(c **and **d) **Clonal T-47D cell lines with *mdm2 *shRNA or control vector were treated with 2 μg/ml doxycycline (DOX) for three days to induce shRNA expression, followed by 10 nM estrogen (E2) for five days in the presence of DOX. **(c) **Western blot analysis of Mdm2, p53, p21 and Actin protein levels from whole cell lysates. **(d) **Number of cells was determined by Guava Viacount assay. A total of 10,000 cells were seeded at beginning of treatments.

To determine if the ability of estrogen to activate cell proliferation occurred strictly through inhibition of wild-type p53 we generated, and examined, MCF-7 cells with inducible shRNA to p53. Reduction of both p53 and p21 protein levels were seen following induction of the *p53 *shRNA (Figure 5a). The addition of estrogen robustly promoted MCF-7 cell proliferation while the knockdown of p53 in the absence of estrogen gave barely any proliferative increase (Figure 5b). Moreover, no further increase of the estrogen-mediated proliferation was achieved by knockdown of p53 (Figure 5b). This suggests that estrogen signaling promotes growth through more pathways than simply blocking p53. Importantly, estrogen's impact strongly depended on Mdm2, suggesting that the Mdm2 influence could also have a p53-independent role.

To further test the p53-independent role of Mdm2 on estrogen signaling we generated inducible shRNA clones from the cell line T-47D that has mutant p53, a G/G SNP309 genotype and is estrogen receptor positive. Induction of shRNA to *mdm2*, but not the vector control, resulted in a decrease in Mdm2 protein without an increase in p21 (Figure 5c, compare lanes 1-2 and 5-6). The addition of estrogen caused a robust increase of Mdm2 that was partially decreased by shRNA induction (Figure 5c, compare lane 3 to lane 4). The addition of estrogen reduced the p21 protein levels (Figure 5c, lanes 3-4 and 7-8) and *mdm2 *shRNA induction did not rescue this reduction (Figure 5c, lane 4). Moreover, the addition of estrogen did not decrease oncogenic mutant p53 (in fact a slight increase was observed). Due to the decreased p21 following estrogen treatment, but the lack of increased p21 following *mdm2 *shRNA induction, it is unclear if Mdm2 is directly degrading p21 in T-47D cells. Importantly, estrogen promoted T-47D cell growth and the depletion of Mdm2 decreased this growth promoting effect (Figure 5d). Estrogen induced growth was not reduced by induction of the vector control. Therefore a p53-independent role of Mdm2 for activation of T-47D cell proliferation was demonstrated.

### Combination of Mdm2 knockdown and etoposide treatment improves growth inhibition of breast cancer cells

**Figure 6:**
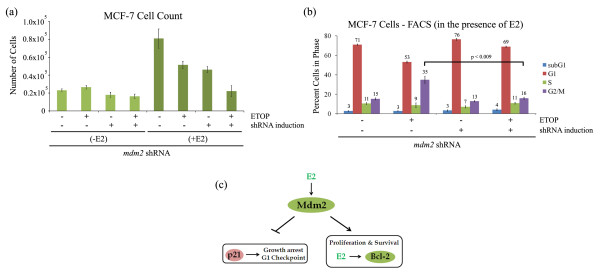
**Combination knockdown of Mdm2 with etoposide treatment improves growth inhibition of breast cancer cells**. Clonal MCF-7 cell line with *mdm2 *shRNA was treated with 2 μg/ml doxycycline (DOX) for three days to induce shRNA expression, followed by 10 nM estrogen (E2) for five days and 50 μM etoposide (ETOP) in the presence of DOX. **(a) **Number of cells was determined by Guava Viacount assay. 10,000 cells were seeded at beginning of treatments. **(b) **Fluorescence activated cell sorting (FACS). Cells were harvested, fixed in 30% ethanol, and cellular DNA was stained with propidium iodide. **(c) **A model illustrating that Mdm2 plays a central role in estrogen-mediated breast cancer cell proliferation.

Because Mdm2 knockdown decreased estrogen-mediated cell proliferation we reasoned that depletion of Mdm2 in MCF-7 cells, in combination with the chemotherapeutic drug etoposide would demonstrate a more robust cell cycle checkpoint response than etoposide alone. We examined the cell proliferation of MCF-7 cells with inducible shRNA to *mdm2*. Estrogen activated proliferation was inhibited to the same extent by either etoposide or induced shRNA to *mdm2 *(Figure 6a). The combination of estrogen and induced shRNA improved the growth inhibition of the estrogen treated cells (Figure 6a). Proliferation without estrogen addition was very low. We examined the number of cells in an estrogen promoted growth phase following treatment with etoposide or with etoposide and knockdown of Mdm2. When Mdm2 was present etoposide treatment caused the cells to pile up in the G2/M cell cycle stage but when shRNA was induced to knockdown Mdm2 the G2 population was reduced and the cells shifted to a more G1 arrest like state (Figure 6b). This G1 checkpoint observed in the *mdm2 *shRNA MCF-7 cells with etoposide and Mdm2 knockdown correlated with increased expression of p21 (Figure 4a).

## Discussion

Estrogen receptor α (ERα) positively regulates growth and development of various tissues, and promotes increased proliferation of breast cancer cells [10]. Based on emerging data, the delicate balance between the opposing functions of p53 and ERα appears to be disrupted in breast cancer cells that over-express the Mdm2 oncogene. Soon after Mdm2 was discovered, ERα was shown to associate with high levels of Mdm2 in breast tumors [7,43,44]. In addition, the estrogen-dependent increase in Mdm2 has been associated with p53 and ERα recruitment to the *mdm2 *gene promoter [8,18]. Our current work has addressed the central dogma of the relationship between Mdm2 upregulation by estrogen and its direct influence on wild-type p53 protein function and breast cancer cell proliferation.

We studied the mechanism by which estrogen might influence the p53 pathway in breast cancer cells with wild-type *p53 *by determining p53 and Mdm2 protein levels and the trans-activation properties of p53 in the MCF-7 (*mdm2 *T/G SNP309) breast cancer cell line. We also examined the influence of estrogen on isogenic MCF-7 cell lines with inducible ("tet-on") short-hairpin RNA to knockdown *p53 *or *mdm2*. We observed that when MCF-7 cells were treated with estrogen, the Mdm2 protein level increased; however unlike in the central p53-Mdm2 dogma, the p53 protein level did not decrease (but slightly increased). Interestingly the ability of p53 to activate transcription was decreased by estrogen and this was relieved by knockdown of Mdm2. The increase in Mdm2 protein in the presence of estrogen was in agreement with previously published data, however, the sustained p53 protein stability with increased Mdm2 has not been previously reported and suggests a higher level of complexity for the Mdm2 oncogenic targets. The negative auto-regulatory feedback loop that exists between p53 and Mdm2 has been shown to be disrupted in cells that carry the G allele in the *mdm2 *gene promoter (SNP309) [15]. The trans-activation ability of p53 varies with the nature of p53-activating stimuli, the cell type and the duration of the activation signal [45,46]. Our data implicates estrogen and ERα as variables that can decrease the trans-activation ability of p53.

In a recent study of gene expression profiles that co-cluster with ERα in breast tumors, it was shown that *puma *is among the genes that are down-regulated after estrogen treatment [47]. Estrogen has been shown to inhibit apoptosis in MCF-7 cells by inducing *bcl-2 *[12]. We observed that estrogen increased proliferation potentially by blocking cell cycle checkpoints. It was interesting that we saw a coordinated up-regulation of Bcl-2 and down-regulation of PUMA protein levels in MCF-7 cells suggesting a need to signal for the inhibition of apoptosis during this increased proliferation. Estrogen-derived oxidants cause DNA damage by oxidative stress and DNA adduct formation [48-50] that could signal for apoptosis. It is possible that the DNA-damaging effects of estrogen in combination with suppression of multiple cell cycle checkpoints set the stage for cancer cells to emerge from cell populations sustaining DNA damage. It is highly likely that estrogen acts in a number of coordinated ways to block cell cycle checkpoints through the p53 and Rb/E2F pathways. Estrogen induces transient cyclical DNA methylation of active promoters that leads to transcription inhibition by changing the histone code [51,52]. Estrogen inhibits resveratrol-activated p53 in MCF-7 cells in part by interfering with post-translational modifications of p53 which are essential for p53-dependent DNA binding and consequent stimulation of downstream pathways [53]. Additionally, the estrogen-mediated increase in Mdm2 protein might lead to p300/CREB transcription co-activators ubiquitination and degradation that would result in reduced acetylation of p53 [54,55]. The *puma *gene is regulated by p300 [56]. Importantly, ERα can bind to p53 directly and repress p53 transcription activation [13,14]. In addition to p53, p73 has been shown to activate *puma *expression [57], therefore it is possible that ERα and Mdm2 inhibit p73 transactivation. Estrogen has been shown to up-regulate Myc [37] and the *puma *gene contains E boxes for Myc binding adjacent to the location of the p53 binding site [58]. This coordinated binding of Myc and p53 or its family members could have implications for the inhibition of *puma *transcription. However, the cooperation of Myc with Mdm2 may have even greater implications for tumor promotion through cross-talk with the RB/E2F pathway as well as the p53 pathway.

We have addressed the impact of estrogen on Mdm2 signal transduction that is both p53-dependent and p53-independent. When either siRNA or shRNA was used to knockdown Mdm2 in MCF-7 cells, the p53 protein level did not increase, but the p53 target gene *p21 *was up-regulated, suggesting activation of p53 transcriptional activity. We demonstrated that knockdown of Mdm*2 *inhibited estrogen-induced proliferation of the MCF-7 cell line. While estrogen promoted MCF-7 cell proliferation, knockdown of wild-type p53 in MCF-7 cells did not. Moreover, knockdown of Mdm2 in MCF-7 cells inhibited cell proliferation to the same extent as the DNA damaging agent etoposide and in combination with etoposide it provoked a robust G1 arrest. Taken together these data suggest that estrogen provokes both a p53-independent and a p53-dependent role for Mdm2 activating the growth of MCF-7 cells. As further evidence for the p53-independent role of Mdm2 in estrogen mediated proliferation, we demonstrated that knockdown of Mdm*2 *inhibited estrogen-induced proliferation of the mutant p53 containing cell line T-47D. While estrogen treatment of T-47D cells resulted in reduced p21 protein the knockdown of Mdm2 in T-47D cells did not increase p21 protein as it did in MCF-7 cells. Recently many breast cancer cell lines were classified as a subtype called senescent cell progenitors (SCPs), which associates with cellular senescence following loss of ERα expression and increased expression of p21 [59]. MCF-7 and T-47D cells fall into the SCP subtype. For the SCP subtype activation of ERα by estrogen protects the cells from senescence. It would be interesting to determine if knockdown of Mdm2 would induce senescence of SCP subtype breast cancers. Combination therapies involving re-activation of checkpoint pathways blocked by Mdm2 (by decreasing Mdm2 protein) may increase the efficacy of killing ERα+ breast cancers. A p53-independent role of Mdm2 has been documented to confer TGFβ resistance in human mammary epithelial cells [60].

The proliferative advantage conferred by estrogen was observed for both the MCF-7 breast cancer cells and the T-47D breast cancer cells. Moreover, we reproducibly observed a more robust influence of estrogen on MCF-7 cells than on T-47D cells. It is possible that this is due to the fact that T-47D cells have mutant p53 and therefore estrogen would only influence p53-independent signal transduction. In MCF-7 cells, with wild-type p53, estrogen can impact both p53-dependent as well as p53-independent pathways. The estrogen proliferative advantage conferred to MCF-7 cells was visible in 3D culture as well as in 2D culture. MCF-7 cells have a mass-like morphology in matrigel, that is similar in size to the acinus, but has a filled lumen indicative of an intermediate aggressive breast cancer cell morphology [42]. We observed that MCF-7 cells formed large mass-like structures in 3D and that these structures were replaced with small structures when Mdm2 was knocked down, suggesting that Mdm2 may be important for invasive behavior of breast cancer cells. Further studies on the role of Mdm2 in aggressive metastatic cells need to be conducted.

## Conclusions

Herein we demonstrated that estrogen modestly inhibits p53 transactivation of target genes in ERα+ breast cancer cells but robustly blocks a proliferative checkpoint pathway through the upregulation of Mdm2. We have demonstrated that estrogen uses an Mdm2-mediated pathway to provoke cell proliferation and that this pathway facilitates p53-independent signal transduction in addition to the capacity to inhibit wild-type p53 function. Therefore, in ERα+ breast cancer Mdm2 may antagonize multiple proliferative checkpoints in a way reminiscent of viral oncogenes.

## Abbreviations

AP1: activator protein 1; Bcl-2: B-cell lymphoma 2; cDNA: complimentary DNA; ChIP: chromatin immunoprecipitation; DAPI: 4',6-diamidino-2-phenylindole; DMSO: Dimethyl sulfoxide; DOX: doxycycline; E2: estrogen; ERα: estrogen receptor alpha; ERα+: estrogen receptor alpha positive; ETOP: etoposide; FACS: fluorescence-activate cell sorter; FBS: fetal bovine serum; FITC: Fluorescein isothiocyanate; Mdm2: mouse double minute 2; p53-RE: p53 response element; PBS: phosphate-buffered saline; PUMA: p53-upregulated mediator of apoptosis; qRT-PCR: quantitative reverse transcriptase polymerase chain reaction; RIPA: radio immunoprecipitation buffer; rtTA: reverse tetracyline-dependent transactivator; shRNA: short hairpin RNA; siRNA: small interfering RNA; SNP309: single nucleotide polymorphism at position 309; SP1: specificity protein 1; SCP: senescent cell progenitors.

## Competing interests

The authors declare that they have no competing interests.

## Authors' contributions

JB and AB wrote the manuscript. JB conceptualized and designed the study. AB designed and carried-out the experiments. JB, AB, AP and NK worked on vector-shRNA cloning and generation of clonal MCF-7 and T-47D cell lines with inducible *mdm2 *and *p53 *shRNA constructs. KES contributed to study conception and design, and set-up of estrogen treatment conditions. All authors read, critiqued and approved the final manuscript.
